# Pressure-modulated dural repair during unilateral biportal endoscopic spine surgery: technique description and preliminary feasibility report

**DOI:** 10.3389/fsurg.2026.1955075

**Published:** 2026-10-14

**Authors:** Bon-Jour Lin, Hao-Yuan Hung, Kuan-Yin Tseng, Dueng-Yuan Hueng, Yuan-Hao Chen, Da-Tong Ju, Hsin-I. Ma, Ming-Ying Liu, Hong-Jaan Wang

**Affiliations:** 1 Graduate Institute of Medical Sciences, College of Medicine, National Defense Medical University, Taipei, Taiwan; 2 Department of Neurological Surgery, Tri-Service General Hospital, National Defense Medical University, Taipei, Taiwan; 3 Graduate Institute of Pharmacology, College of Pharmacy, National Defense Medical University, Taipei, Taiwan; 4 Faculty of Pharmaceutical Sciences, University of British Columbia, Vancouver, BC, Canada

**Keywords:** dural repair, durotomy, inlay graft, irrigation pressure, unilateral biportal endoscopic surgery

## Abstract

**Background:**

The clinical management of incidental durotomy during the performance of endoscopic spine surgery presents a significant clinical challenge. Instrument maneuverability is limited, and the unique aqueous environment that introduces complications secondary to the intradural influx of irrigation fluid. The purpose of this study was twofold: first, to describe in detail a novel water-pressure modulation technique for collagen matrix inlay graft placement for incidental durotomy repair during unilateral biportal endoscopic spine surgery (UBES); and second, to evaluate the efficacy of this technique in three specific clinical cases, focusing on its feasibility and safety.

**Methods:**

This retrospective study was conducted on clinical cases involving patients who underwent UBES for degenerative lumbar spine pathology from September 2022 to September 2024, based on a review of medical records. Surgical documentation was rigorously examined to ascertain occurrence of incidental durotomy during UBES. The clinical characteristics associated with the durotomy were systemically documented alongside the specific repair technique employed.

**Results:**

A total of three incidental durotomies were identified from 91 UBES procedures performed, resulting in an incidence rate of 3.29%. In each case, the incidental durotomy was successfully repaired through the modulation of irrigation pressure to facilitate the placement of a collagen matrix inlay graft. No patients experienced postoperative cerebrospinal fluid leak or required revision surgery.

**Conclusions:**

Analysis of these clinical cases demonstrated the technical feasibility and preliminary safety of irrigation pressure modulation for collagen matrix inlay graft placement for incidental durotomy repair during UBES. However, further studies with a larger sample size are required to confirm these findings.

## Introduction

Recently, the use of endoscopic spine surgery for the management of degenerative spinal disorders has become significantly more common. In contrast to microscopic surgery, endoscopic spine surgery provides various benefits, such as diminished tissue trauma and expedited recovery periods (1, 2). However, it also introduces distinct challenges due to the reliance on a single-handed instrument technique and the hydrodynamic environment in which it operated (3, 4).

Incidental durotomy is the dominant complication encountered in spinal surgery, with reported incidence rates ranging from 1.8% to 14% (5, 6). The appropriate management of incidental durotomy is imperative to mitigate potential complications, which include cerebrospinal fluid (CSF) leaks, intracranial hypotension, wound infection, and, in severe cases, central nervous system infection. Incidental durotomy during endoscopic spine surgery creates a significantly more precarious situation; the intradural influx of irrigation fluid through a durotomy aperture has the potential to lead to elevated intracranial pressure, seizures, and even irreversible neurological deficits (7–11). During unilateral biportal endoscopic spine surgery (UBES), a surgeon is limited to using a single hand to manipulate their instruments. Meanwhile, turbulence generated by endoscope irrigation fluid exacerbates the challenges of durotomy repair. To date, only a few studies have proposed management strategies for repairing incidental durotomy during endoscopic spine surgery (12–16). The decision-making process in such cases is influenced by the dimensions of the durotomy and the nature of the condition, specifically dependent on the occurrence of root incarceration or herniation through the dural defect. Open repair is recommended for patients presenting with complex defects and root incarceration, whereas endoscopic repair is suggested simple defects.

The optimal strategy to avoid complications involves the prompt and effective repair of identified durotomies during endoscopic spine surgery (9, 10). This study presents a technical and feasibility report. Our objective was twofold: to describe in detail a novel water-pressure modulation technique for collagen matrix inlay graft placement for the repair of incidental durotomies during UBES; and second, to preliminarily evaluate the efficacy of this technique in clinical cases, as measured by dural repair success rates and the need for revision surgery.

## Materials and methods

This retrospective study was conducted on clinical cases and approved by the Institutional Review Board of Tri-Service General Hospital on January 14, 2026 (Approval Number: B202605019). Medical records were examined for a cohort of consecutive patients who underwent UBES for the treatment of degenerative lumbar spine pathology during the period from September 2022 to September 2024. Explicit eligibility criteria were applied for patient selection. The inclusion criteria comprised: 1. Diagnosis of degenerative lumbar spine pathology refractory to conservative management; 2. Undergoing primary UBES intervention. Patients were excluded from the study if they presented with: 1. A documented history of lumbar trauma, spinal infection, or systemic malignancy; or 2. A history of previous lumbar spine surgery at the index level. To eliminate inter-operator variability, all UBES procedures were performed by a single neurosurgeon (first author) possessing three years of specialized clinical experience in endoscopic spine surgery.

Relevant data pertaining to each patient, including diagnosis, surgical methodology, intraoperative findings, and preoperative/postoperative symptoms were systemically reviewed. Surgical recordings were analyzed to identify instances of incidental durotomy occurring during the UBES procedure. When incidental durotomy was identified, the size, anatomical location and repair technique used were comprehensively recorded.

### Operative technique

#### Step 1: Decompression and visualization

During UBES, continuous irrigation was maintained using a pressure-controlled pump system (10k ®, ConMed Linvatec, Florida, United Stated) set at a target pressure of 30 mmHg. Upon initial identification of the incidental durotomy, pump-assisted irrigation was switched to gravity-based irrigation positioned at 30 cm above the operation level, providing a maximum hydrostatic pressure of 30 cmH_2_O (approximately 22.1 mmHg). And rapid decompression around the site was performed to facilitate a clear visualization and enable subsequent manipulation (Figure 1A).

**Figure 1:**
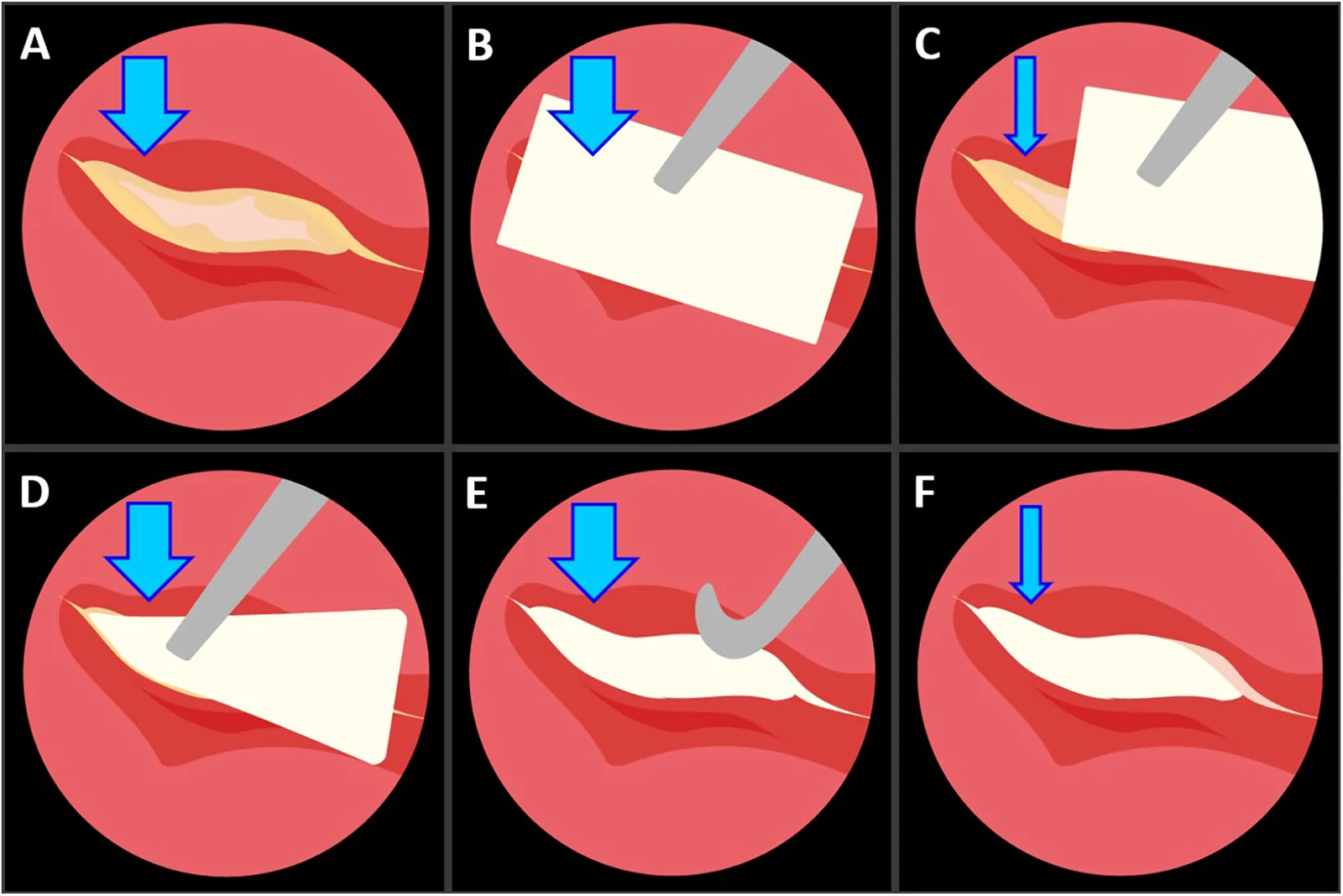
The steps of applying irrigation pressure modulation to place a collagen matrix inlay graft for the repair of incidental durotomy during unilateral biportal endoscopic spine surgery. **(A)** The surgical perspective and operational environment surrounding the durotomy were established under standard irrigation pressure (indicated by the thick blue arrow). **(B)** A sizable DuraGen was chosen to ensure comprehensive coverage. **(C)** The irrigation pressure was reduced (indicated by the thin blue arrow) to mitigate the impact of fluid turbulence on the dura mater and DuraGen graft. **(D)** After the insertion of one corner of the DuraGen under reduced irrigation pressure, the irrigation pressure was elevated. **(E)** The increase in hydrostatic pressure produced a positive differential pressure relative to the intradural space, thereby promoting the assimilation of residual DuraGen into the durotomy. **(F)** Upon the conclusion of the procedure, the irrigation pressure was lowered again to assess the positioning of the implanted DuraGen.

#### Step 2: Graft selection and grafting

Upon determination of the dimensions and morphology of the durotomy, a fragment of collagen matrix graft (DuraGen, Integra LifeSciences Corporation, Plainsboro, New Jersey, United States) multiple times greater in size than the dural defect was chosen (Figure 1B) (17–19).

#### Step 3: Lowing irrigation pressure and graft insertion

Prior to the placement of DuraGen, the gravity-based irrigation pressure was lowered from 30 to 10 cm above the operation level, providing a maximum hydrostatic pressure of 10 cmH_2_O (approximately 7.4 mmHg), to minimize fluid turbulence and potential subsequent morphological alteration of the dura mater and durotomy. Under relatively low irrigation pressure, one edge of the DuraGen was inserted into the dural defect using microscopic instruments (Figure 1C).

#### Step 4: Increasing irrigation pressure for graft fixation

After inserting one edge of the DuraGen into the durotomy, the gravity-based irrigation reservoir height was restored from 10 to 30 cm above the operation level. The irrigation fluid pressure was elevated (Figure 1D), producing a positive differential pressure in relation to the intradural space, which secured the floating DuraGen and facilitated the incorporation of remaining graft into the durotomy (Figure 1E).

#### Step 5: Graft stability verification and procedure resumption

Once the DuraGen was placed, the gravity-based irrigation reservoir was lowered to 10 cm above the operation level to ascertain whether the graft would remain in the predetermined position (Figure 1F). If the DuraGen was securely placed, the operational gravity-based irrigation reservoir was restored to 30 cm above the operation level to advance the incomplete surgical procedure.

#### Step 6: Specific conditional management

- cauterization-assisted dural shrinkage

In cases of large durotomy, bulging of the DuraGen may occur due to intradural pressure. To resolve this, a bipolar radiofrequency device (ArthroCare Coblation System, ArthroCare Corporation, Austin, Texas, United States) was used to cauterize the dura mater surrounding the durotomy. This intervention served to shrink the durotomy and prevent the DuraGen from bulging outward. Controlled thermal shrinkage tightened the rim around the dural defect, providing a snug, stable aperture that mechanically retained the inlay graft in its intended subdural position.

- placement of overlay DuraGen graft

If the durotomy was located on the dorsal aspect of the thecal sac, an additional piece of DuraGen was positioned as overlay. One edge of the overlay graft was secured under the inferior margin of the lamina, thereby preventing displacement caused by fluid turbulence. The overlay DuraGen graft acted as a buttress to reinforce graft retention and maintain direct apposition of the inlay Duragen graft against the inner dural surface.

### Postoperative radiological protocol

Immediate postoperative magnetic resonance imaging (MRI) was scheduled for patients experiencing CSF leak-related signs of newly developed neurological deficits. Conversely, in the absence of such expressions, follow-up MRI was scheduled at 3 months postoperatively in accordance with national health insurance guidelines.

## Results

Between September 2022 and September 2024, our team conducted 91 UBES procedures across 84 patients (45 male and 39 female; clinical data in Table 1), including unilateral laminotomy for bilateral decompression (ULBD), interlaminar lumbar discectomy, transforaminal lumbar interbody fusion, and paraspinal approaches. Within this overall cohort, the incidence rate of dural injury was 3.29% (3/91). All three instances of occurred in the thecal sac during removal of the ligamentum flavum; two were located on the dorsal aspect, while one was situated on the lateral aspect. Each of these three incidental durotomies was addressed using the described inlay DuraGen placement technique with modulation of the irrigation pressure. Clinical records pertaining to the incidental durotomies and corresponding managing strategies for the three patients are delineated in Table 2.

**Table 1:** Clinical characteristics of included surgical procedures.

Characteristics	Number
Total number of patients	84
Demographics	
Sex	
Male	45
Female	39
Age (years)	65.60 ± 17.40
Follow-up (months)	15.87 ± 7.16
Diagnosis	
Spinal canal stenosis	33
HIVD	20
recurrent HIVD	4
Spondylolisthesis	15
Foraminal stenosis	19
UBES procedure	
Chosen approach	91
Bilateral decompression	32
Decompression with discectomy	23
Lumbar interbody fusion	17
Extraforaminal approach	19
Levels of decompression	
one level	73
two levels	15
three levels	3

**Table 2:** Clinical characteristics and intraoperative features of the observed dural injuries.

Age(y)	Sex	Diagnosis	UBES procedure	Location of durotomy	Durotomy size	Cause of injury	Repairment technique
83	F	Spinal canal stenosis, L4-5	ULBD	Thecal sac, lateral aspect	4mm	Rongeur	Inlay DuraGen graft; dural shrinkage
66	M	Spinal canal stenosis, L5-1	ULBD	Thecal sac, dorsal aspect	3mm	Rongeur	Inlay and overlay DuraGen grafts
66	F	Spinal canal stenosis, L3-4	ULBD	Thecal sac, dorsal aspect	8mm	Direct traction	Inlay and overlay DuraGen grafts; dural shrinkage

UBES, unilateral biportal endoscopic spine surgery; ULBD, unilateral laminotomy for bilateral decompression.

After surgery, a drainage catheter was introduced to mitigate the risk of postoperative hematoma, and each patient was instructed to remain in a recumbent position for one day before any ambulation the following day. No patient experienced discomfort associated with incidental durotomy in the postoperative phase. Notably, the three subjects who experienced incidental durotomies exhibited remarkably homogenous clinical characteristics and defect profiles. Because of the strictly uniform and standardized water-pressure modulation technique conducted by the same surgeon, consistent technical feasibility and preliminary safety was achieved, with no incidence of postoperative CSF leak or need for revision surgery. All three patients experienced an uneventful postoperative recovery, and the 3-month follow-up MRI revealed no pseudomeningocele or extradural CSF collection.

### Illustrative cases

#### Case number one

An 83-year-old female presented with neurological claudication, persisting for six months. Magnetic resonance image (MRI) of the lumbar spine disclosed the presence of central canal stenosis at the L4–5 level, necessitating the execution of an UBES–ULBD. An incidental durotomy measuring 4 mm in length was noted on the lateral aspect of the thecal sac during excision of the ligamentum flavum with a Kerrison rongeur. The durotomy was repaired by inlay DuraGen placement and cauterization-assisted durotomy shrinkage (Figure 2).

**Figure 2:**
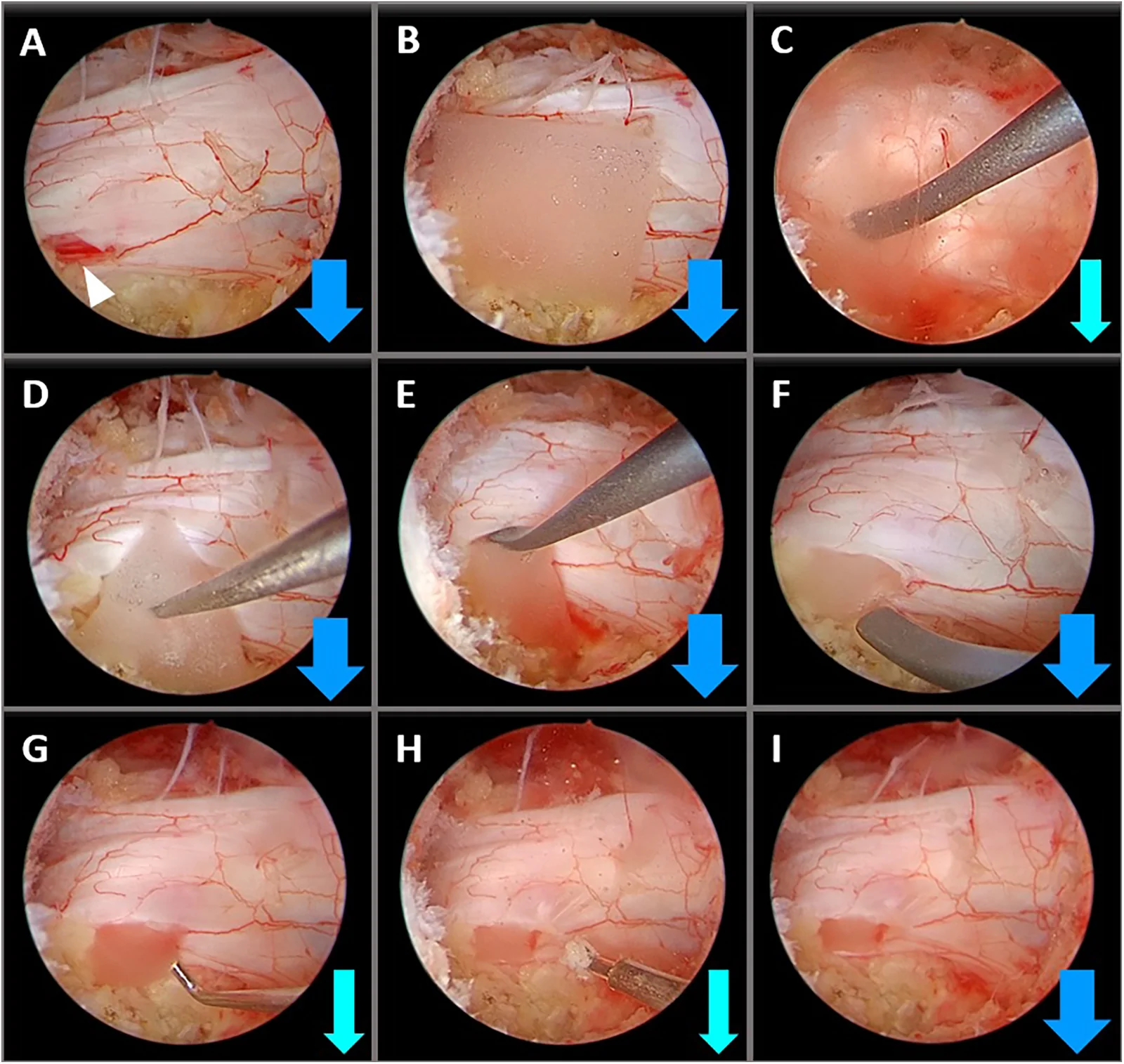
Intraoperative endoscopic photos of surgical procedure (patient 1). **(A)** A 4-mm incidental durotomy, as indicated by the arrowhead, was noted on the lateral aspect of the thecal sac under a gravity-based irrigation reservoir of 30 cm above the operation level, denoted by the thick blue arrow. **(B)** A single patch of DuraGen was selected, of a size exceeding that of the durotomy. **(C)** The irrigation reservoir was subsequently lowered to 10 cm above the operation level, as signified by the thin blue arrow, allowing for the insertion of one corner of DuraGen through the durotomy. **(D–F)** The irrigation fluid reservoir was readjusted to 30 cm above the operation level, facilitating the repositioning of DuraGen to the intended location with the aid of the augmented irrigation pressure. **(G)** The irrigation reservoir was once again lowered to 10 cm above the operation level, at which point a protrusion of the DuraGen was noted. **(H)** The ArthroCare device was employed to cauterize the dura mater surrounding the durotomy to induce shrinkage. **(I)** Final photographic documentation of the durotomy repair was conducted.

### Case number two

A 66-year-old male complained of left leg pain lasting for three months. MRI of the lumbar spine revealed lateral recess stenosis at the L5–S1 level, requring UBES–ULBD. When dissecting the upper insertion of the ligamentum flavum with a Kerrison rongeur, an incidental durotomy measuring 3 mm in length was noted on the dorsal aspect of the thecal sac. The durotomy was rectified through the implantation of inlay DuraGen placement, accompanied by overlay DuraGen coverage (Figure 3).

**Figure 3:**
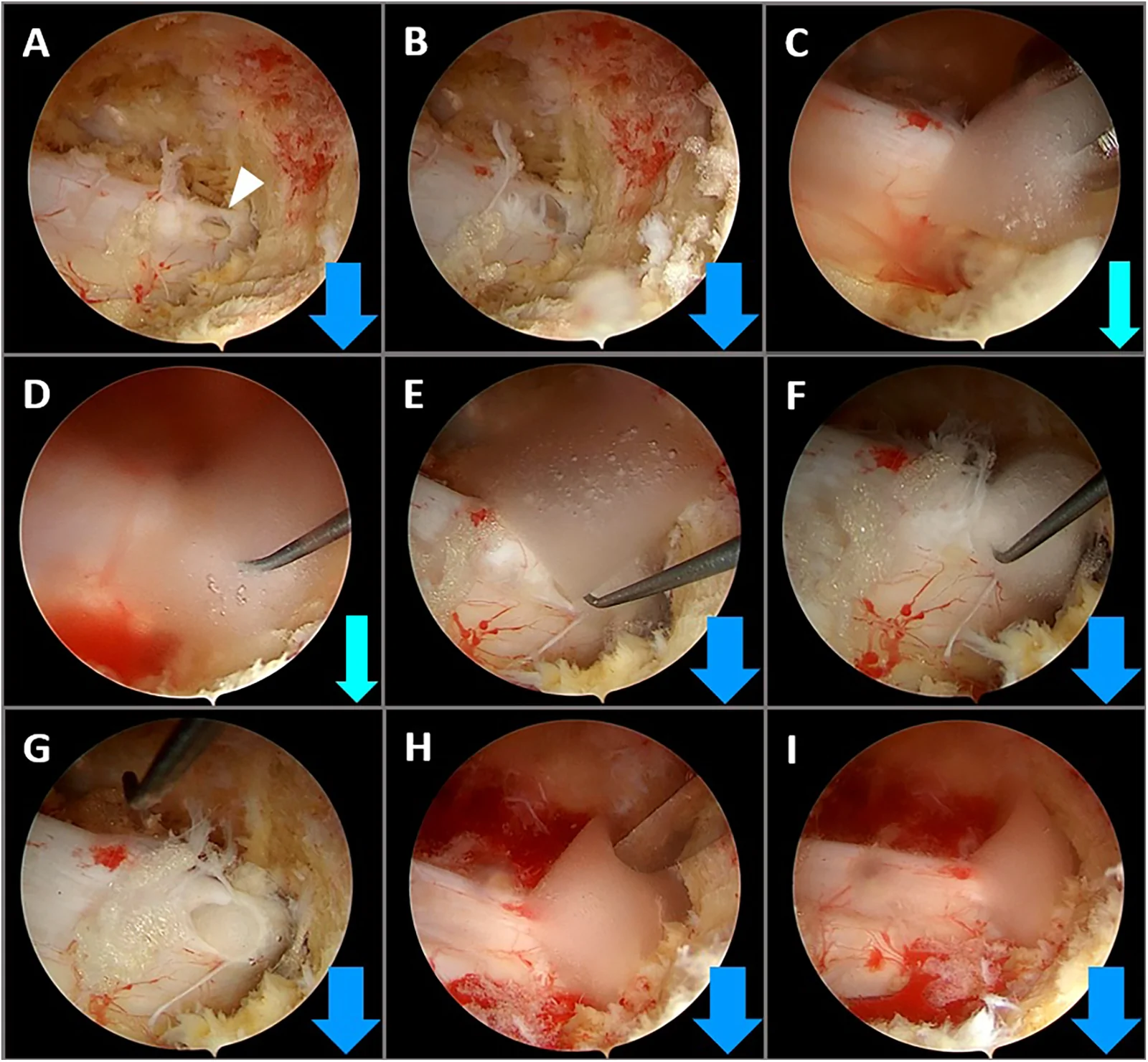
Intraoperative endoscopic photos of surgical procedure (patient 2). **(A)** A 2-mm incidental durotomy (indicated by the white arrowhead) was observed in the dorsal aspect of the thecal sac under a gravity-based irrigation reservoir of 30 cm above the operation level (indicated by the thick blue arrow). **(B)** A magnified examination of the durotomy revealed the presence of an intact arachnoid membrane. **(C)** A large-sized DuraGen was selected for deployment as an inlay graft. **(D)** The irrigation reservoir was lowered to 10 cm above the operation level (indicated by the thin blue arrow), thereby facilitating the introduction of one corner of DuraGen through the durotomy. **(E,F)** The irrigation reservoir was subsequently restored to 30 cm above the operation level, allowing for the repositioning of the DuraGen to the specified site with the aid of enhanced irrigation pressure. **(G)** The DuraGen graft was completely introduced with the support of augmented irrigation fluid pressure. **(H)** To further improve the repair, additional overlay DuraGen coverage was applied and secured to the inferior margin of the lamina. **(I)** The final photograph documenting the durotomy repair was captured.

### Case number three

A 66-year-old female experienced severe low back pain over a period of three months. MRI of the lumbar spine showed central canal stenosis at the L3–4 level, leading to UBES-ULBD intervention. An incidental durotomy measuring 8 mm in length was detected while excising the central segment of the ligamentum flavum with forceps and subsequent traction of the central dura fold. Management of the durotomy involved the placement of inlay DuraGen, ArthroCare-assisted durotomy shrinkage, and application of overlay DuraGen coverage (Figure 4).

**Figure 4:**
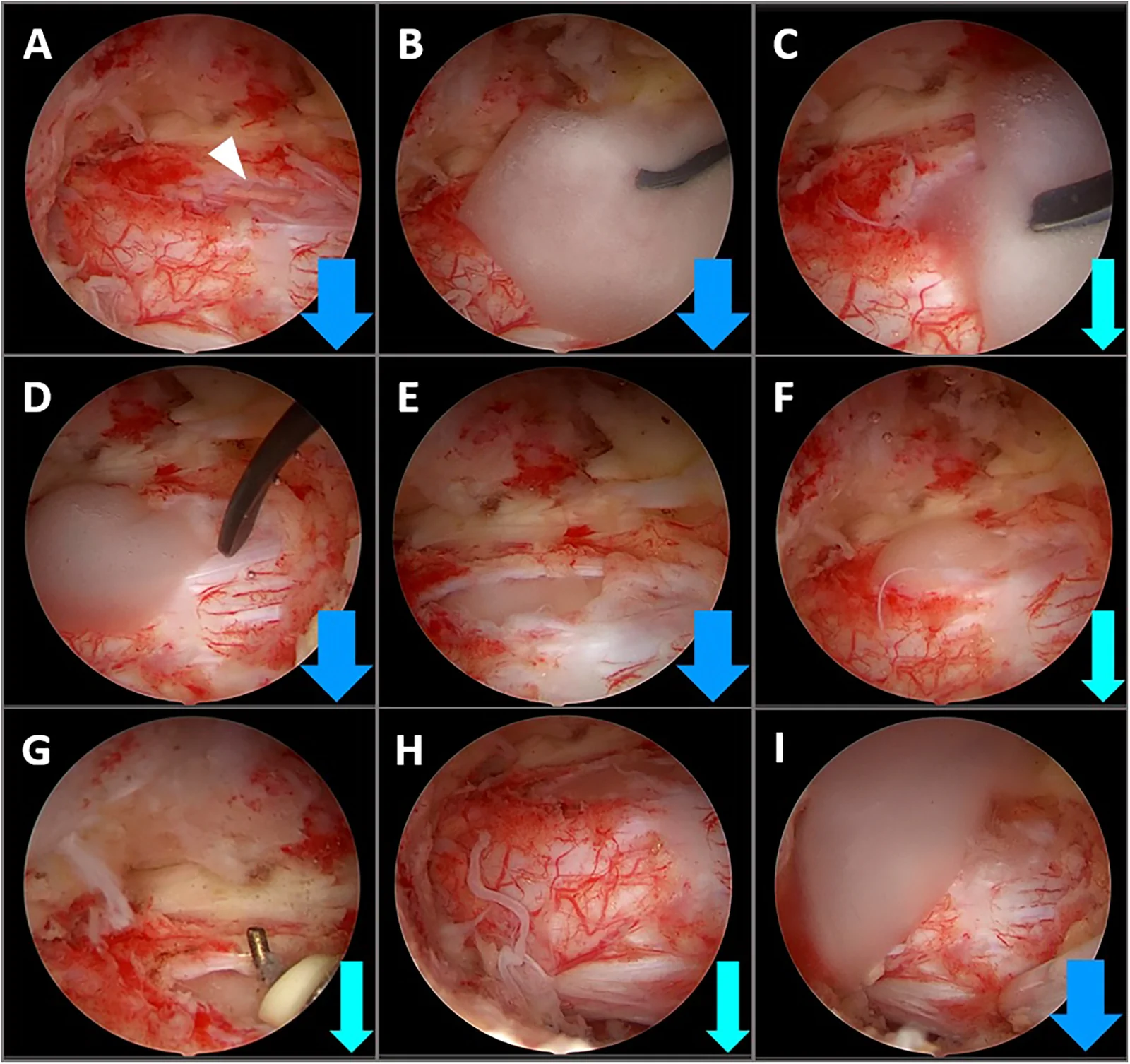
Intraoperative endoscopic photos of surgical procedure (patient 3). **(A)** An 8-mm incidental durotomy (indicated by the arrowhead) was observed on the dorsal aspect of the thecal sac under a gravity-based irrigation reservoir of 30 cm above the operation level (as denoted by the thick blue arrow). **(B)** A large-sized DuraGen was selected for use as an inlay graft. **(C)** The irrigation reservoir was lowered to 10 cm above the operation level (as denoted by the thin blue arrow), thereby permitting the introduction of one corner of DuraGen through the durotomy. **(D,E)** The irrigation reservoir was subsequently elevated to 30 cm above the operation level, enabling the repositioning of DuraGen to the designated site with the assistance of the enhanced irrigation pressure. **(F)** The irrigation reservoir was once more lowered to 10 cm above the operation level, and a protrusion in the DuraGen was noted. **(G)** The ArthroCare device was utilized to cauterize the dura mater adjacent to the durotomy, facilitating dura shrinkage. **(H)** Conclusive photographic documentation of the durotomy repair was performed. **(I)** An overlay DuraGen was applied and secured to the inferior margin of the lamina.

## Discussion

Among the methodologies employed in endoscopic repair, the two principal approaches are direct suturing and the placement of blocking patches. Polypropylene sutures and non-penetrating clips have been documented as efficacious for endoscopic direct suturing; however, their success is contingent upon the structural integrity of the dura mater (20, 21). This is particularly pertinent for patients exhibiting a fragile dura mater, as external force applied to the dural edges may result in an increased size of the durotomy. Conversely, to overcome the technical challenges of direct suturing, a variety of materials have been developed as blocking patches for managing incidental durotomy in endoscopic spine surgery, with favorable outcomes. These materials include autologous grafts (such as fat or muscle), absorbable gelatin compressed sponges, and collagen patches containing fibrinogen and thrombin (22–25). Although these patch-based approaches obviate the need for delicate suturing in confined spaces, each material carries distinct degrading rates, swellability, and adhesion properties. A primary concern associated with patch placement is that bulky or non-transparent implants may inadvertently obstruct endoscopic visualization and hinder subsequent dissection procedures. Furthermore, procedural displacement or migration of these patches can cause persistent CSF leak and potentially exert an unwanted focal mass effect on adjacent neural structures.

The clinical use of DuraGen has been reported in the context of open spine surgery for the purpose of rectifying incidental durotomy (17, 18). Its implementation in the realm of endoscopic spinal surgery has also been corroborated by a more recent study (24). The theory supporting this approach posits that intradural pressure will exert force on the inlay DuraGen against the inner dura wall, thereby self-sealing the dural defect and effectively constraining CSF egress. In clinical practice, once continuous irrigation is suspended, successful repair must verify that the inlay matrix remains fully apposed to and completely bridges the durotomy edge. However, within a hydrous environment, the ultimate positioning of the implanted DuraGen remains uncertain once irrigation ceases, as the fixation efficacy of fibrin sealant under such conditions is questionable.

In the present investigation, we implemented adjustments to the irrigation pressure to facilitate the placement of a large-sized, inlay DuraGen for managing incidental durotomy during UBES. Upon identifying the incidental durotomy, the irrigation modality was intentionally converted from a pressure-controlled pump system to gravity-based irrigation system. Although pressure-controlled pump system provides a more stable pressure profile under normal conditions—contingent on maintaining uncompromised outflow—it lacks the fine granularity for ultra-low pressure delivery in our setup (minimum setting: 30 mmHg). In the event of dural violation, switching to a gravity-based irrigation system ensures an intrinsic physical ceiling. Lowering the irrigation reservoir to 10–30 cm above the operation level generated ultra-low hydrostatic pressures (approximately 7.4–22.1 mmHg), which minimized fluid turbulence and prevented excessive intrathecal fluid infusion. The physical and biological rationale for this sutureless inlay technique relies on a dynamic balance of pressure and tissue integration. Biomechanically, once irrigation is discontinued, the inherent hydrostatic intradural pressure drives the highly conformable DuraGen outward, pressing it firmly against the margins of the dural defect. This outward pressure, combined with the natural surface tension and friction between the dural tissue and the collagen matrix, effectively prevents CSF outflow without requiring mechanical fixation. Biologically, the porous collagen matrix of DuraGen acts as an endogenous scaffold, rapidly absorbing local blood and proteins to form a cohesive clot, which eventually promotes native dural fibroblast migration and regenerative closure (19). This approach exhibits high target specificity for the unique microenvironment of UBES. Unlike open surgery, the confined working space of UBES renders conventional microsurgical dural suturing technically prohibitive. By temporarily modulating irrigation pressure during the insertion of the DuraGen, we can stabilize the dural defect and counteract early CSF gushing, creating a temporary dry-like pocket that allows a large DuraGen to be unfurled and positioned precisely over the target defect. Regarding clinical feasibility, this sutureless method offers distinct translational advantages. Given the relative simplicity and rapid nature of the procedure, it is capable of rapidly reducing potential influx of irrigation fluid into the intradural space. The implanted inlay DuraGen is unlikely to obscure the operative field, thereby allowing the operator to advance with the wider procedure. Furthermore, this technique does not require manipulation of the dura mater; this is particularly advantageous in instances where the dura mater is fragile, as excessive manipulation may lead to an increased defect. However, there remains lack of understanding regarding the efficacy of this technique in cases of large durotomy, especially in the lateral region of the thecal sac. The additional maneuvers (cauterization-assisted dural shrinkage and placement of overlay grafting) are not intended as standalone repair barriers; rather, they serve strictly as adjunctive maneuvers for positional stabilization to prevent migration, folding, or displacement of the primary inlay graft during high-flow pulsation of hydrostatic environment.

The most effective strategy for preventing incidental durotomy is through proactive measures (14, 26, 27). The improper use of surgical instruments and inadequate dissection practices are the primary causes of incidental durotomy. Therefore, careful selection of appropriate instruments and thorough dissection are essential practices in mitigating the risk of incidental durotomy during UBES. In this technical and feasibility report, we have described the successful implementation of a novel water-pressure modulation technique for repairing incidental durotomy during UBES. Beyond detailing the surgical steps, this report also highlights the clinical utility and preliminary feasibility of the technique. However, the study is primarily limited by the small number of cases that required this water-pressure modulation technique, and its preliminary nature. The feasibility study lacked a comparative control group, such as historical controls or patients undergoing standard collagen matrix inlay placement without pressure modulation. Consequently, our results regarding the clinical application of this technique must be considered preliminary, and we cannot definitively determine whether it offers a significant clinical advantage over alternative repair methods.

Finally, several limitations must be acknowledged. First, the small sample size in this preliminary series inherently limits the generalizability of our findings. Second, because our technique represents a multimodal approach combining dynamic irrigation pressure modulation, the deployment of an oversized inlay collagen matrix, and physiological tissue adherence, the independent contribution of irrigation pressure control cannot be fully isolated. Future prospective comparative trials or biomechanical stimulation studies with larger sample size are warranted to validate the specific benefits and superiority of this approach.

## Conclusions

Prompt repair of incidental durotomy is crucial for minimizing the intradural reflux of irrigation fluid during endoscopic spine surgery. While analysis of these clinical cases demonstrated the technical feasibility and preliminary safety of our pressure-modulated dura repair technique, further studies with a larger sample size are required to confirm these preliminary findings.

## Data Availability

The original contributions presented in the study are included in the article/Supplementary Material, further inquiries can be directed to the corresponding author.
